# Major CD4 T-Cell Depletion and Immune Senescence in a Patient with Chronic Granulomatous Disease

**DOI:** 10.3389/fimmu.2017.00543

**Published:** 2017-05-11

**Authors:** Adriana S. Albuquerque, Susana M. Fernandes, Rita Tendeiro, Rémi Cheynier, Margarida Lucas, Susana L. Silva, Rui M. M. Victorino, Ana E. Sousa

**Affiliations:** ^1^Instituto de Medicina Molecular, Faculdade de Medicina, Universidade de Lisboa, Lisbon, Portugal; ^2^Centro de Imunodeficiência Primárias de Lisboa, Lisbon, Portugal; ^3^Hospital de Santa Maria, Centro Hospitalar Lisboa Norte, Lisbon, Portugal; ^4^Cytokines and Viral Infections, Immunology Infection and Inflammation Department, Institut Cochin, INSERM, U1016, Paris, France; ^5^CNRS, UMR8104, Paris, France; ^6^Université Paris Descartes, Paris, France

**Keywords:** primary immunodeficiency, chronic granulomatous disease, genetic phagocytic defect, reactive oxygen species, CD4 T-cell lymphopenia, immune senescence, gut mucosa, interleukin-17

## Abstract

Chronic granulomatous disease (CGD) results from primary defects in phagocytic reactive oxygen species (ROS) production. T-cell evaluation is usually neglected during patients’ follow-up, although T-cell depletion has been reported in CGD through unknown mechanisms. We describe here a 36-year-old patient with X-linked CGD with severe CD4 T-cell depletion <200 CD4 T-cells/μl, providing insights into the mechanisms that underlie T-cell loss in the context of oxidative burst defects. In addition to the typical infections, the patient featured a progressive T-cell loss associated with persistent lymphocyte activation, expansion of interleukin (IL)-17-producing CD4 T-cells, and impaired thymic activity, leading to a reduced replenishment of the T-cell pool. A relative CD4 depletion was also found at the gut mucosal level, although no bias to IL-17-production was documented. This immunological pattern of exhaustion of immune resources favors prompt, potentially curative, therapeutic interventions in CGD patients, namely, stem-cell transplantation or gene therapy. Moreover, this clinical case raises new research questions on the interplay of ROS production and T-cell homeostasis and immune senescence.

## Introduction

Chronic granulomatous disease (CGD) is the most common primary immunodeficiency affecting phagocytes. It is due to genetic defects in nicotinamide adenine dinucleotide phosphate (NADPH) oxidase (1, 2), leading to impaired reactive oxygen species (ROS) production by monocytes and neutrophils, defective microorganism clearance, and chronic inflammation (1–4). Reduced T-cell numbers were reported in an American CGD cohort, although the underlying mechanisms remain unclear (5). The defective ROS production by myeloid cells indirectly contributes to the T-cell loss through the promotion of inflammation (1, 2). On the other hand, T-cells have been shown to harbor NADPH (6), and T-cell intrinsic defects in ROS production are associated with disturbances in T-cell effector differentiation and regulatory function (6–11). Notably, CGD patients feature expansion of the interleukin (IL)-17-producing T helper cell subset, which is thought to contribute to their heightened immune activation state (12). T-cell alterations in susceptibility to apoptosis have also been described (13), as well as impairments in autophagy (14), which may also impact on T-cell development and function.

We report here long-lasting severe CD4 lymphopenia in a 36-year-old patient with CGD that allowed us to investigate the pathways involved in T-cell production and peripheral homeostasis in the context of markedly impaired ROS production. Our findings are consistent with an overall scenario of immunological exhaustion and are of foremost clinical relevance in the context of the increased life expectancy of CGD patients, as well as to the ongoing debate regarding aggressive early therapeutic intervention (15–17).

## Case Report

X-linked CGD was only diagnosed at the age of 13 (G897A mutation at the extreme end of exon 8 of the *CYBB* gene), although the patient had a clinical history typical of this disorder, namely, neonatal *Staphylococcus aureus* sepsis, *Salmonella enteritidis* sepsis (at 7 years of age), multiple liver abscesses due to *S. aureus* requiring partial hepatectomy (at 10 years of age), and recurrent suppurative lymphadenitis (axillary and cervical), as well as subcutaneous abscesses. ROS production was undetectable using a flow cytometry-based oxidative burst assay (<1% positive monocytes or neutrophils; BD Biosciences). Prophylactic antimicrobials were started with an effective control of major infections, except *Pseudomonas aeruginosa* keratoconjunctivitis (at age 16).

**Figure 1:**
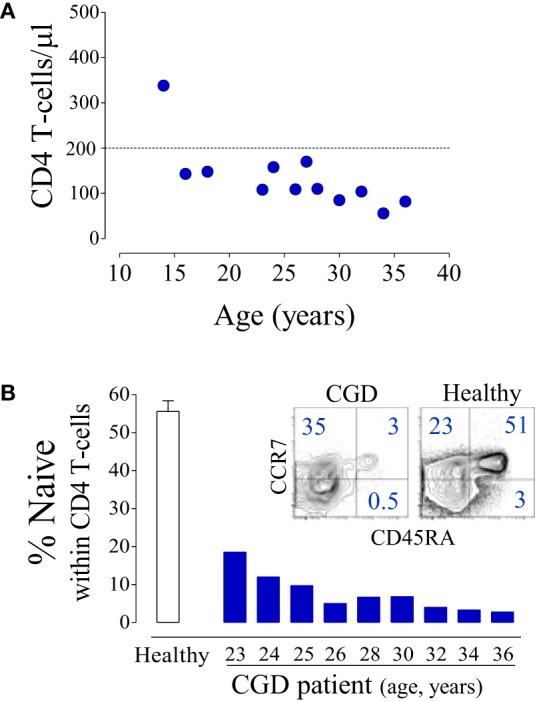
**Major CD4 T-cell depletion in a chronic granulomatous disease (CGD) patient**. **(A)** Longitudinal CD4 T-cell counts. **(B)** Proportion of naïve (CD45RA+CD27+) CD4 T-cells in the CGD patient; open bar represents mean ± SEM of healthy subjects (*n* = 25), and dot plots illustrate CCR7/CD45RA expression within CD4 T-cells.

The patient featured a progressive loss of T-cells with sustained CD4 T-cell counts <200 cells/μl (Figure 1A) and an inverse CD4/CD8 ratio (varying between 0.6 and 0.09).

**Figure 2:**
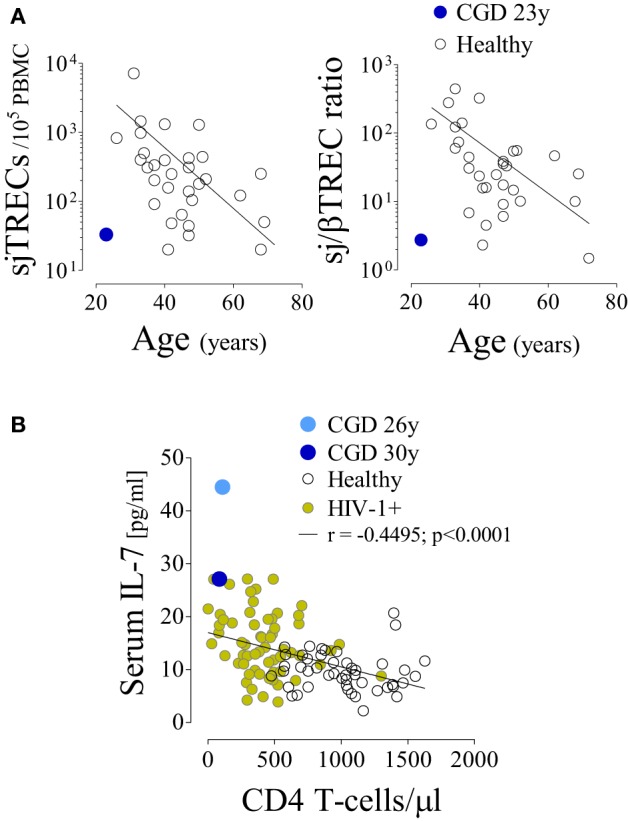
**Impaired T-cell production despite increased circulating interleukin (IL)-7 levels in a chronic granulomatous disease (CGD) patient**. **(A)** Signal joint T-cell receptor rearrangement excision circle (sjTREC) frequency and sj/βTREC ratio in total T-cells in relation to age in the CGD patient and healthy subjects. **(B)** IL-7 levels and CD4 counts in the CGD patient (blue), healthy subjects (black, *n* = 47), and untreated HIV-1-infected individuals (green, *n* = 61); each dot represents one individual/time point. *P*-value and Spearman’s correlation coefficient are shown.

In parallel with the T-cell depletion, the phenotypic analysis revealed marked loss of naïve cells within both CD4 (Figure 1B) and CD8 T-cells (6.2% of total CD8 T-cells, 100 cells/μl at 36 years of age). This was in agreement with an impaired replenishment of the T-cell pool by recently produced cells. Indeed, we found evidence of compromised thymopoiesis *via* the quantification of by-products of T-cell receptor (TCR) rearrangement that are generated during thymic T-cell development [signal joint (sj) and DβJβ TCR rearrangement circles, T-cell receptor rearrangement excision circle (TREC)] and progressively decline during age-associated thymus involution. Both sjTREC frequency and the sj/βTREC ratio, which are considered to reflect intra-thymic precursor T-cell proliferation and directly correlate with thymic output (18, 19), were markedly low for the patient’s age (Figure 2A). Of note, reduced thymic activity was observed despite the levels of IL-7, an essential homeostatic cytokine, being highly enhanced (Figure 2B), even in comparison with those of untreated HIV-1-infected individuals. Thus, impaired thymopoiesis seemed to significantly contribute to naïve T-cell loss. The risks inherent to the reduced thymic activity in conjunction with the patient’s age contributed to the decision to not undergo hematopoietic stem-cell transplantation (HSCT).

**Figure 3:**
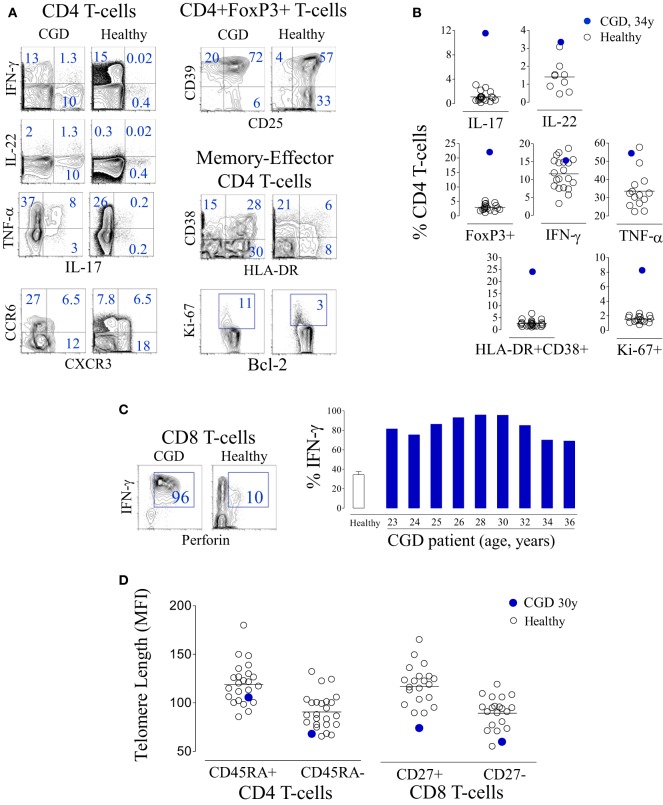
**T-cell activation and terminal differentiation in a chronic granulomatous disease (CGD) patient**. **(A)** Representative dot plots of the analysis of cytokine production and chemokine expression within CD4 T-cells, regulatory T-cell-associated markers within CD4+FoxP3+ cells, as well as activation and cycling markers within memory-effector CD4 T-cells from the CGD patient at 36 years of age and a representative age-matched healthy control. **(B)** Frequency of interleukin (IL)-17-, IL-22-, IFN-γ-, or TNF-α-producing cells, as well as the frequency of FoxP3+, HLA-DR+CD38+, or Ki-67+ cells within the CD4 subset, in the peripheral blood of the CGD patient and healthy subjects. **(C)** Representative dot plots showing the co-expression of IFN-γ and perforin within CD8 T-cells in the CGD patient and an age-matched control, and a graph showing the frequency of IFN-γ-producing cells within CD8 T-cells of the CGD patient at different ages and of healthy adults (bar represents mean ± SEM, *n* = 24). **(D)** Telomere length of naïve (CD45RA+) and memory (CD45RA−) CD4 T-cells as well as CD8 T-cells defined according to CD27 expression from the CGD patient and controls.

Naïve CD4 T-cell loss was accompanied by upregulation of activation markers and by increased frequency of cycling cells (Figure 3A). The analysis of cytokine production by T-cells revealed an effector differentiation profile with a significant production of pro-inflammatory cytokines, particularly IL-17 (Figure 3B), as has been previously reported (12, 20). Of note, the majority of IL-17-producing T-cells were CD4+ (96%). There was also an increased frequency of CD4 T-cells producing IL-22 (Figure 3B), of which 42% concomitantly produced IL-17. Notably, there was a parallel overrepresentation of regulatory T-cells (Treg) expressing high levels of CD25 and CD39, markers associated with suppressive function (Figures 3A,B). CD8 T-cells featured an activated and terminally differentiated phenotype, as illustrated by their high levels of perforin and IFN-γ production (Figure 3C). In agreement with these findings, both CD4 and CD8 T-cells, irrespective of their degree of differentiation, featured markedly reduced telomere length, further supporting persistent immune stimulation and increased cell cycling in parallel with reduced *de novo* T-cell replenishment (Figure 3D).

**Figure 4:**
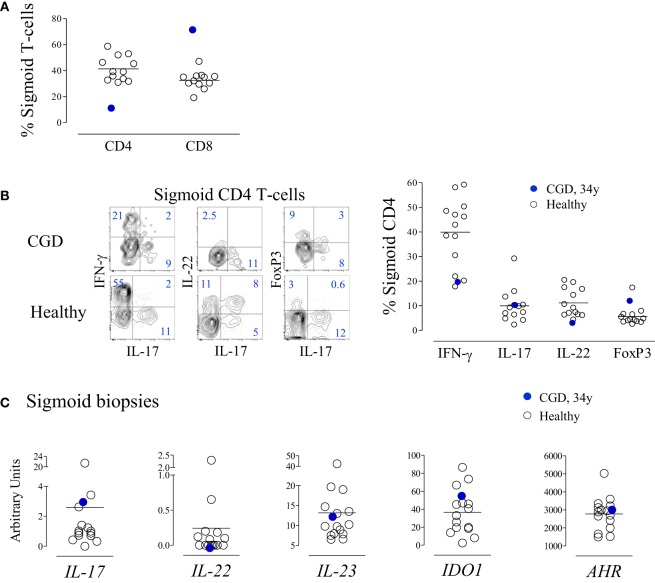
**T-cell subsets in the gut mucosa of the chronic granulomatous disease (CGD) patient**. **(A)** Frequency of CD4 and CD8 T-cells within CD3+ lymphocytes in cell suspensions of sigmoid biopsies from the CGD patient at 34 years of age and from healthy subjects (*n* = 12). **(B)** Representative dot plots of the expression of IFN-γ, interleukin (IL)-17, IL-22, and FoxP3 within sigmoid CD4+ cells from the CGD patient and an age-matched control; the graph indicates the frequency of IFN-γ-, IL-17-, and IL-22-producing cells, as well as FoxP3+ cells within sigmoid CD4+ cells from the CGD patient and healthy subjects. **(C)** *IL-17, IL-22, IL-23, IDO1*, and *AHR* mRNA levels of expression; total RNA extracted from sigmoid biopsies of the CGD patient and healthy controls and mRNA levels expressed in relative units, normalized to the mean *C*_t_ levels of *GAPDH* and *r18S*. Each dot represents one individual. Bars represent mean of values obtained in healthy controls.

At 34 years of age, colonoscopy was performed due to an episode of prolonged diarrhea, with watery stool four to five times a day without mucus or blood, with no apparent microbial cause, accompanied by hypoalbuminemia without other evidence of exudative enteropathy or significant malabsorption. The gastrointestinal symptoms subsided spontaneously after 4 weeks. The mucosa was macroscopically normal with an overall preserved structure, with areas of mild inflammatory infiltrates with lymphoid aggregates in the gut biopsies. Cell suspensions from sigmoid biopsies were analyzed by flow cytometry, and patient data were compared with those of healthy subjects submitted to routine colon cancer screening colonoscopy. The CGD patient featured a decreased frequency of CD4+ cells within total T-lymphocytes in the sigmoid mucosa (Figure 4A) and a very low CD4/CD8 ratio (0.14), although we were unable to quantify the absolute counts in the histological material to confirm the CD4 loss. Importantly, in contrast to the circulating CD4 subset, the relative proportions of cells producing IL-17, IL-22, or IFN-γ, as well as FoxP3-expressing Treg, within sigmoid CD4 cells were within the range of healthy subjects (Figure 4B). Moreover, there were no alterations in the levels of expression of genes implicated in the regulation of these populations, namely, *IL-17, IL-22, IL-23, IDO1*, and *AHR*, in the sigmoid biopsies (Figure 4C). To the best of our knowledge, this is the first report of T-cell imbalances illustrating the occurrence of CD4 depletion at the gut mucosa in CGD. The apparent lack of Th17 expansion at the mucosal level warrants further investigation, given the known role of IL-17 in inflammatory bowel disorders (21, 22) and the importance of gut pathology in CGD patients.

## Methods

### Patient and Controls

Longitudinal data obtained from a 36-year-old Caucasian patient with X-linked CGD were compared with results obtained from healthy donors (23, 24) and a previously described cohort of untreated HIV-1-infected individuals (25), which featured a wide CD4 T-cell loss range. All individuals gave written informed consent. The study was approved by the ethical board of the Faculty of Medicine of University of Lisbon.

### Cell Isolation

Fresh peripheral blood mononuclear cells (PBMCs) were isolated from heparinized venous blood immediately after collection by standard Ficoll-Hypaque density gradient centrifugation (Amersham Pharmacia Biotech, Uppsala, Sweden). Approximately 7–10 sigmoid biopsies collected from the patient and from individuals performing colonoscopy in the context of routine clinical follow-up with macroscopically normal mucosa (24) were digested with collagenase B (10 mg/ml; Roche, Penzberg, Germany), at 37°C, immediately after collection; mechanically macerated; and the lymphocytes separated using a Percoll gradient (GE Healthcare, Uppsala, Sweden; 40% over 80%), as previously described (24).

### Flow Cytometry Studies

Lymphocyte phenotypic analysis was performed in whole blood after surface staining with monoclonal antibodies (mAbs) (26). The following anti-human mAbs were used: FITC-conjugated HLA-DR (clone L243) and CD45RA (clone HI100); PE-conjugated CD4 (clone RPA-T4), CD27 (clone O323), and CD38 (clone HB7); PerCP-conjugated CD4 (clone RPA-T4), CD8 (clone RPA-T8), and CD3 (clone OKT3); and APC-conjugated CD25 (clone 2A3), purchased from either BD Biosciences (San Jose, CA, USA) or eBiosciences (San Diego, CA, USA). Intracellular staining for FoxP3 (clone PCH101 from eBiosciences), Bcl-2 (clone 124 from Dako), Ki-67 (clone B56 from BD Biosciences), or perforin (clone 27-37 from BD Biosciences) was performed in PBMC using eBiosciences’s kit after surface staining, as previously described (26). At least 100,000 events were acquired on FACSCalibur or LSRFortessa cytometers (BD Biosciences), and data were analyzed using FlowJo software (Tree Star Inc., Ashland, OR, USA). The absolute numbers of lymphocyte subsets were calculated by multiplying their representative frequency by the absolute lymphocyte count obtained at the clinical laboratory.

### Cytokine Production

Cytokine production by PBMC or mucosal lymphocytes was assessed at the single-cell level after 4-h culture with 50 ng/ml phorbol 12-myristate 13-acetate (Sigma-Aldrich, St. Louis, MO, USA) plus 500 ng/ml ionomycin (Calbiochem; Merck Millipore) in the presence of 10 µg/ml brefeldin A (Sigma-Aldrich), as previously described (27). The following anti-human mAbs were used for intracellular staining: IFN-γ (clone 4S.B3), IL-17 (clone eBio64DEC17), TNF-α (clone MAb11), and IL-22 (clone 22URTI), all from eBiosciences.

### TREC Analysis

Signal joint and DJ TREC analyses were conducted as described (18, 19). Briefly, triplicate multiplex PCR amplification for sjTREC, DJβ1TRECs (Dβ1-Jβ1.1 to 1.6), or DJβ2TRECs (Dβ2-Jβ2.1 to 2.7), together with the CD3γ chain, was performed on lysed PBMC. TREC and CD3γ quantifications were then performed using a LightCycler™ in independent experiments, with the same first-round serial dilution standard curve. This highly sensitive nested quantitative PCR assay allowed detection of one copy in 10^5^ cells for any excision circle. The sj/βTREC ratio was calculated [sjTREC/10^5^cells/(DJβ1TRECs/10^5^cells + DJβ2TRECs/10^5^cells)], as described (18).

### IL-7 Quantification

Serum IL-7 levels were measured using Quantikine HS ELISA kit (R&D Systems, Minneapolis, MN, USA), according to the manufacturer’s instructions (25). All samples were assayed in duplicate.

### Telomere Length Measurement by Flow Fluorescent *In Situ* Hybridization Coupled to Flow Cytometry (Flow-FISH)

Telomere length was assessed by Flow-FISH technique on thawed PBMCs, as previously described (23, 28). Briefly, after surface staining with CD45RA-FITC, CD27-FITC, and biotin-conjugated CD4 or CD8, PBMCs were fixed for 30 min at 4°C with 1 mM *bis*(sulfosuccinimidyl)suberate. The reaction was quenched using 1 ml of 50 mM Tris pH7.2 for 20 min at room temperature. After washing in PBS, followed by hybridization buffer containing 70% formamide, 20 mM Tris-HCl, 1% BSA, and 1.5 M NaCl, cells were incubated with the protein nucleic acid telomeric probe (C3TA2)3 conjugated to Cy5. After heating for 10 min at 82°C, samples were left to hybridize for 60–90 min, washed in post-hybridization buffer followed by PBS, and analyzed immediately by flow cytometry. All samples were run in triplicate.

### mRNA Extraction from the Gut and Transcripts Expression

One sigmoid biopsy was stored in RLT buffer (Qiagen, Valencia, CA, USA) immediately post collection. RNA was extracted using Allprep RNA/DNA mini kit (Qiagen), and 250 ng was used to synthesize cDNA (SuperScript III Reverse Transcriptase; Life Technologies), as described (24). Expression levels of IL-17A, IL-22, IL-23, indoleamine 2,3-dioxygenase (IDO) 1, and aryl hydrocarbon receptor (AHR) were measured after pre-amplification with TaqMan PreAmp Master Mix, using TaqMan gene expression assays with an Applied Biosystems 7500 Fast Real-Time PCR System (all from Life Technologies), as previously described (24). Results were expressed as Δ*C*_t_ normalized to the medium *C*_t_ levels of glyceraldehyde 3-phosphate dehydrogenase (GAPDH) and r18S.

## Discussion

Our finding of sustained severe long-term CD4 T-cell lymphopenia in X-linked CGD calls attention to the occurrence of major T-cell imbalances in the context of burst oxidative defects and raises the possibility of T-cell immunological exhaustion. To our knowledge, this is the first report of CD4 depletion at the gut mucosal level in CGD, in agreement with peripheral blood data. Therefore, increasing effort should be devoted to the understanding of the mechanisms underlying T-cell loss in CGD and their clinical implications.

This immune senescent profile has been considered of immunological risk for infections and shown to be an independent predictor of death in aged subjects (29). Moreover, it resembles the profile of immunosenescence documented in individuals thymectomized early in life during corrective cardiac surgery (26, 30, 31).

Our case suggests that CGD-associated T-cell depletion may worsen with age, which further supports early therapy potentially curative interventions such as HSCT and gene therapy (2, 4, 15–17, 32).

Of note, the persistent immune activation of our patient featured an AIDS-like immunological profile (33–35). CGD is typically associated with an inflammatory and hyper-activated state (3, 32, 36, 37). In CGD mouse models, inflammation has been linked to a defective production of the immunosuppressive molecule l-kynurenine (20), a tryptophan catabolism intermediate generated by the enzyme IDO, leading to increased IL-17 production. However, recent data showed that CGD patients have preserved IDO function in circulating leukocytes (38, 39). Nevertheless, our patient featured a relative expansion of IL-17-producing CD4 T-cells as compared to other CD4 T-cell subsets, in agreement with previous reports (3, 12, 20), which likely contributed to the hyperactivated state. Notably, the Th17 overrepresentation was not found at the gut mucosa level, a finding that warrants further investigation given the importance of gut pathology in CGD and the known role of IL-17 in inflammatory bowel disorders (21, 22).

The observed massive IL-7 levels may also contribute to the exaggerated immune stimulation. The increase in IL-7 levels in lymphopenic settings is thought to be mostly related to lack of consumption due to the reduction in available targets (25, 40). However, in our case, they were much above those found in untreated HIV-infected individuals with comparable T-cell depletion (25). This finding raises the possibility of IL-7 being partially produced as an acute-phase response in the liver, as reported upon toll-like receptors signaling in mouse models (41).

Of note, in spite of the high levels of IL-7, there was marked impairment of thymopoiesis and a consequent defective replenishment of the naïve compartment, as attested by the reduced sj/βTREC ratio and T-cell telomere length.

Interestingly, it has also been suggested that T-cells have intrinsic defects in ROS production in CGD, which may compromise their thymic development as well as their peripheral survival and function (6–11).

## Concluding Remarks

Long-lasting severe CD4 lymphopenia may occur in CGD, likely due to both persistent immune activation and impaired T-cell production. This profile of early immune senescence represents a further argument in favor of timely curative therapeutic interventions in CGD, namely, stem-cell transplantation or gene therapy. Additionally, in a broader perspective, our results open new areas of research regarding the interplay of ROS production and T-cell homeostasis and immune senescence.

## Ethics Statement

The study was approved by the ethical board of the Faculty of Medicine of University of Lisbon. All individuals gave written informed consent.

## Author Contributions

AA, RV, and AS designed the study; AA, SF, RT, RC, and SS performed research; ML, SS, and RV collected clinical data; AS supervised the study; and AA and AS wrote the paper.

## Conflict of Interest Statement

The authors declare that the research was conducted in the absence of any commercial or financial relationships that could be construed as a potential conflict of interest.
